# Poldip2/Nox4 Mediates Lipopolysaccharide-Induced Oxidative Stress and Inflammation in Human Lung Epithelial Cells

**DOI:** 10.1155/2022/6666022

**Published:** 2022-01-30

**Authors:** Yueguo Wang, Zhenxing Ding, Youhui Tu, Xu Wu, Wenying Zhang, Shuang Ji, Jilong Shen, Li Zhang, Huimei Wu, Guanghe Fei

**Affiliations:** ^1^Department of Respiratory and Critical Care Medicine, The First Affiliated Hospital of Anhui Medical University, Hefei, 230022 Anhui, China; ^2^Department of Emergency Medicine, The First Affiliated Hospital of USTC, Division of Life Sciences and Medicine, University of Science and Technology of China, Hefei, 230001 Anhui, China; ^3^Department of Pathogen Biology and Provincial Laboratories of Pathogen Biology and Zoonoses, Anhui Medical University, Hefei, 230032 Anhui, China; ^4^The Center for Scientific Research, The First Affiliated Hospital of Anhui Medical University, Hefei, 230022 Anhui, China; ^5^Anhui Geriatric Institute, Department of Geriatric Respiratory and Critical Care, The First Affiliated Hospital of Anhui Medical University, Hefei, 230022 Anhui, China; ^6^Key Laboratory of Respiratory Disease Research and Medical Transformation of Anhui Province, The First Affiliated Hospital of Anhui Medical University, Hefei, 230022 Anhui, China

## Abstract

NADPH oxidase 4 (Nox4) is an important source of reactive oxygen species (ROS) production, and its expression is increased in lipopolysaccharide- (LPS-) stimulated lung epithelial cells. Polymerase *δ*-interacting protein 2 (Poldip2) has been proved to bind Nox4 and participates in oxidative stress and inflammation. However, the role of Poldip2/Nox4 in LPS-induced oxidative stress and inflammation in lung epithelial cells remains unclear. Cell viability was measured via MTT assays. The expression of Poldip2, Nox4, heme oxygenase-1 (HO-1), cyclooxygenase-2 (COX-2), AKT, and p-AKT was detected by Western blotting and/or immunofluorescence. Poldip2 and Nox4 interaction was analyzed via coimmunoprecipitation (Co-IP) assay. NADPH enzymatic activity and production of ROS, prostaglandin E2 (PGE2), tumor necrosis factor-*α* (TNF-*α*), and interleukin-1*β* (IL-1*β*) were assessed simultaneously. The small interfering RNA (siRNA) or plasmid targeting Nox4 was used to downregulate or upregulate Nox4, and the lentiviral vector encoding Poldip2 was used to downregulate or upregulate Poldip2. The present study demonstrated that LPS stimulation significantly increased the protein levels of Poldip2 and Nox4 and proved that Poldip2 interacted with Nox4 proved by Co-IP. Importantly, Poldip2 acted as an upstream regulator of Nox4. The increased expression of Nox4 and COX-2; NADPH enzymatic activity; production of ROS, PGE2, TNF-*α*, and IL-1*β*; and decreased HO-1 expression were significantly suppressed by lentiviral Poldip2 shRNA downregulation but were increased by lentiviral upregulation of Poldip2. Furthermore, inhibiting of PI3K-AKT signaling notably attenuated LPS-induced Poldip2/Nox4 activation. Our study demonstrated that Poldip2 mediates LPS-induced oxidative stress and inflammation via interaction with Nox4 and was regulated by the PI3K-AKT signaling. Targeting Poldip2 could be a beneficial therapeutic strategy for the treatment of ALI.

## 1. Introduction

Lipopolysaccharide (LPS), a major constituent of the cell walls of Gram-negative bacteria, plays a vital role in the development and progression of acute lung injury (ALI) [1]. It can initiate production of biologically active molecules from a variety of cells [2, 3], including inflammatory cytokines and reactive oxygen species (ROS). Mounting evidence suggests that oxidative stress is associated with the progression of ALI [4, 5]. Excessive ROS production overwhelms antioxidant defenses thereby increasing the inflammatory processes and ultimately leading to tissue injury, especially lung epithelial cell damage [6]. The NADPH oxidase family of enzymes is thought to be a major source of ROS under various pathological conditions [7], which consists of seven isozymes, including Nox1–Nox5, DUOX1, and DUOX2 [8]. Importantly, NADPH oxidase 4 (Nox4) is widely expressed in human lung epithelial cells [9]. Previously, studies showed that LPS markedly increased NADPH oxidase activation, including Nox4, and intracellular ROS production in human lung epithelial cells [10] and the mouse preclinical model of ALI [11]. These observations indicate that Nox4-mediated oxidative stress and inflammation may be enhanced by LPS stimulation and implicated in LPS-induced ALI. However, the mechanism of the activation of Nox4 and Nox4-mediating effects during LPS stimulation is not completely understood.

Additionally, polymerase *δ*-interacting protein 2 (Poldip2) (also known as polymerase delta-interacting protein 38 (PDIP38) and mitogenin-1) is a multifunctional protein initially identified as a binding partner of the DNA polymerase *δ* p50 subunit and proliferating cell nuclear antigen (PCNA) [12]. Poldip2 is widely expressed in human primary pulmonary microvascular endothelial cells (HPMECs) [13] and lung epithelial cells [14]. In addition to its role in DNA replication and damage repair [12], Poldip2 served as a protein chaperone and is involved in mitochondrial function and morphology [15, 16], extracellular matrix synthesis [17], and degradation [18], as well as cell cycle progression [19]. Previously, a study has demonstrated that Poldip2 binds Nox4 in a yeast two-hybrid assay [20], it also increases Nox4 enzymatic activity, and positively regulates basal ROS production in vascular smooth muscle cells and HPMECs [13, 20]. In addition, a recent study demonstrated that reduced Poldip2 protects against LPS induced-acute respiratory distress syndrome (ARDS) through suppressing mitochondrial ROS production in a mouse model [13]. Collectively, Poldip2 is a key protein in regulating the progression of ALI but how Poldip2 regulates the expression of Nox4 and enzymatic activity of NADPH oxidases in response to LPS in lung epithelial cells deserves further research.

The aim of this study was at determining the potential specific role of Poldip2, identifying how it regulates the expression of Nox4, enzymatic activity of NADPH oxidases, and Nox4 downstream effects on human lung epithelial cells and to elucidate the potential molecular mechanisms further.

## 2. Materials and Methods

### 2.1. Reagents and Materials

DMEM medium and 1640 medium were purchased from HyClone (HyClone, USA), and fetal bovine serum was purchased from Gibco (Gibco, USA). LPS and LY294002 were purchased from Sigma (Sigma-Aldrich, USA). RIPA lysis buffer, DAPI, rabbit IgG, puromycin, and FITC-conjugated secondary antibody were purchased from Beyotime (Beyotime, China). Primary antibodies of Poldip2, HO-1, COX-2, AKT, p-AKT, and *β*-actin were purchased from Abcam (Abcam, UK). Primary antibodies of Nox4 were purchased from Novus (Novus, USA).

### 2.2. Cell Culture

The human lung adenocarcinoma cell line A549 and human bronchial epithelial cell line Beas-2B were obtained from the Cell Bank of Chinese Academy of Sciences (Shanghai, China). Cells were cultured at 37°C in a humidified atmosphere with 5% CO_2_ in DMEM medium or 1640 medium supplemented with 10% fetal bovine serum, 100 U/mL penicillin, and 100 mg/mL streptomycin, respectively.

### 2.3. Cell Viability Assay

The cell viability was measured by MTT assay. Cells were treated with different doses of LPS (0, 2.5, 5, 10, 15, 20, and 40 *μ*g/mL for A549 cells and 0, 0.25, 0.5, 1, 2, 5, and 10 *μ*g/mL for Beas-2B cells) for 24 h, 10 *μ*L of MTT solution (5 mg/mL) was added to each well for culturing at 37°C for 4 h, and 100 *μ*L of DMSO was added to solubilize formazan crystals. The absorbance at a 570 nm wavelength was measured by a microplate reader (BioTek, USA). The results were expressed as the mean percentage of absorbance in the treatment group versus the control group.

### 2.4. Western Blotting

Cell proteins were extracted using RIPA lysis buffer. Proteins were electrophoresed on 10% SDS-PAGE gel and electrotransferred to polyvinylidene difluoride (PVDF) membranes. The membranes were blocked using 5% skimmed milk at room temperature for 2 h and incubated with primary antibodies for 12 h at 4°C. Horseradish peroxidase-conjugated secondary antibody was added at 37°C for 1 h. In this study, primary antibodies of Poldip2 (1 : 1000), Nox4 (1 : 1000), HO-1 (1 : 1000), COX-2 (1 : 1000), AKT (1 : 1000), and p-AKT (1 : 1000) were used and *β*-actin (1 : 5000) worked as an endogenous control. The bands were detected by enhanced a chemiluminescence (ECL) detection kit (Thermo Fisher Scientific, Barrington, IL, USA). Densitometry for all proteins was normalized against control.

### 2.5. NADPH Enzymatic Activity Assay

NADPH enzymatic activity was measured using a commercially available kit (Genmed Scientifics, Shanghai, China) according to the manufacturer's protocols. Briefly, the supernatant of cell lysates was incubated with oxidized cytochrome c in a quartz cuvette at 30°C for 3 min and then mixed with the Nox substrate (NADPH) and incubated for 15 min. The change of absorbance at 340 nm was monitored by a spectrophotometer. NADPH enzymatic activity was estimated by calculating cytochrome c reduction per min.

### 2.6. Coimmunoprecipitation Assay

For the endogenous interaction assay, cells were cultured in a 10 cm culture dish with 60% confluence and then treated with LPS for 12 h. After lysis and collection of cells, equal amounts of lysates were incubated overnight at 4°C with 3 *μ*g of the primary antibodies of Poldip2 or normal rabbit IgG (isotype controls) and protein G Mag Sepharose (Thermo Fisher Scientific, Waltham, MA, USA). The proteins bound to antibodies were pulled down by protein G beads and subjected to immunoblotting analysis.

### 2.7. Intracellular ROS Assay

Production of intracellular ROS was measured with dichlorodihydrofluorescein diacetate (DCFH-DA) (Beyotime, China) per the manufacturer's instructions. Briefly, the cells were treated with trypsin, incubated with 10 *μ*mol/L DCFH-DA at 37°C for 20 min, and washed with PBS 3 times. Then, cells were rinsed with DMEM and analyzed by flow cytometry (BD Biosciences, Franklin Lakes, NJ).

### 2.8. Determination of PGE2, TNF-*α*, and IL-1*β*

Enzyme-linked immunosorbent assay (ELISA) was used to measure the levels of PGE2, TNF-*α* and IL-1*β* in culture supernatants according to the manufacturer's instructions. The levels of TNF-*α* and IL-1*β* were determined using ELISA kits (absin, China), and PGE2 was determined using ELISA kits (CUSABIO BIOTECH, China).

### 2.9. siRNA-Mediated Downregulation of Nox4

The siRNA targeting Nox4 and negative control siRNA were obtained from the Hanbio Biotechnology Co. Ltd. (Shanghai, China). Cells were seeded and cultured in 6-well plates for 24 h with 30–50% confluence. Cells were transfected with siRNA using lipofectamine 2000 (Invitrogen, Carlsbad, CA, USA) in accordance with the manufacturer's protocols. Nox4 siRNA-transfected cells and negative control siRNA-transfected cells were defined as siNox4 and siCont. After 72 h, the expression of Nox4 and transfection efficiency were detected by Western blotting. The siNox4 sequences are listed as follows: sense: 5′-GCAAUAAGCCAGUCACCAUTT-3′ and antisense: 5′-AUGGUGACUGGCUUAUUGCTT-3′.

### 2.10. Plasmid-Mediated Upregulation of Nox4

The Nox4 plasmid and empty vector were constructed by the Hanbio Biotechnology Co. Ltd. (Shanghai, China). Cells were seeded and cultured in 6-well plates for 24 h with 30–50% confluence. Cells were transfected with Nox4 plasmid or the empty vector by lipofectamine 2000 following the manufacturer's instructions. After 72 h, the expression of Nox4 and transfection efficiency were detected by Western blotting. The primer sequences are listed as follows: forward: 5′-CAAGCTGTGACCGGCGCCTACGAATTCGCCACCATGGCTGTGTCCTGG-3′ and reverse: 5′-ACCCCATCGATGGACCGGTCGGGATCCGCTGAAAGACTCTTTATTGT-3′.

### 2.11. Lentivirus-Mediated Downregulation of Poldip2

A lentivirus vector containing Poldip2-specific shRNA or nonspecific shRNA (as a negative control) was constructed from the Hanbio Biotechnology Co. Ltd. (Shanghai, China). Cells were seeded and cultured in 6-well plates for 24 h with 30–50% confluence. Cells were transfected with a lentivirus vector and incubated for 24 h, and the supernatants were exchanged with fresh culture medium and cultured for 72 h. Stable-transfected cells were isolated using 2 *μ*g/mL of puromycin to screen for puromycin resistance gene production. Poldip2 shRNA-transfected cells and negative control shRNA-transfected cells were defined as shPoldip2 and shCont. After 72 h, the expression of Poldip2 and transfection efficiency were evaluated by Western blotting. The shPoldip2 sequences are listed as follows: top strand: 5′-GATCCGGCGCTACTGTATCCGTTTGGAGAATTCAAGAGATTCTCCAAACGGATACAGTAGCGCCTTTTTTG-3′ and bottom strand: 5′-AATTCAAAAAAGGCGCTACTGTATCCGTTTGGAGAATCTCTTGAATTCTCCAAACGGATACAGTAGCGCCG-3′.

### 2.12. Lentivirus-Mediated Upregulation of Poldip2

Lentivirus upregulating Poldip2 particles and lentivirus upregulating control particles were constructed from the Hanbio Biotechnology Co. Ltd. (Shanghai, China). Cells were seeded and cultured in 6-well plates for 24 h to obtain 30–50% confluence. The lentiviral particles were then added to the wells at a multiplicity of infection (MOI) value of 50 : 1 and cultured with cells. After 24 h, the culture medium was replaced with the fresh complete medium, added 2*μ*g/mL of puromycin, and cultured for an additional 72 h. The puromycin-resistant cells were then isolated. Poldip2-upregulated cells and upregulating control cells were defined as LvPoldip2 and LvCont. The expression of Poldip2 and transfection efficiency were evaluated by Western blotting. The primer sequences are listed as follows: forward: 5′-AGAGGATCTATTTCCGGTGAATTCGCCACCATGGCAGCCTGTACAGCCC-3′ and reverse: 5′-GTCACTTAAGCTTGGTACCGAGGATCCCAGTGAAGGCCTGAGGGTGGT-3′.

### 2.13. Immunofluorescence Analysis

Cells were fixed in 4% paraformaldehyde for 15 min and blocked with 5% goat serum for 2 h at room temperature and then incubated with primary antibodies of Poldip2 and Nox4 at 4°C.After overnight incubation, cells were washed with PBS and incubated with a fluorescein isothiocyanate- (FITC-) conjugated secondary antibody. Nuclei were stained with DAPI. Cells were viewed with an inverted LSM880 confocal laser-scanning microscope (Carl Zeiss, Jena, Germany) with blue and green channels.

### 2.14. Statistical Analysis

All data analyses were performed using SPSS 23.0 software (SPSS Inc., Chicago, IL, USA). The results are expressed as mean ± SD. Comparisons among groups were determined using Student's *t*-test or one-way ANOVA followed by Tukey's post hoc test. *P* < 0.05 was considered statistically significant.

## 3. Results

### 3.1. LPS Increased the Protein Expression of Poldip2 and Nox4 in Lung Epithelial Cells in a Dose-/Time-Dependent Manner

**Figure 1:**
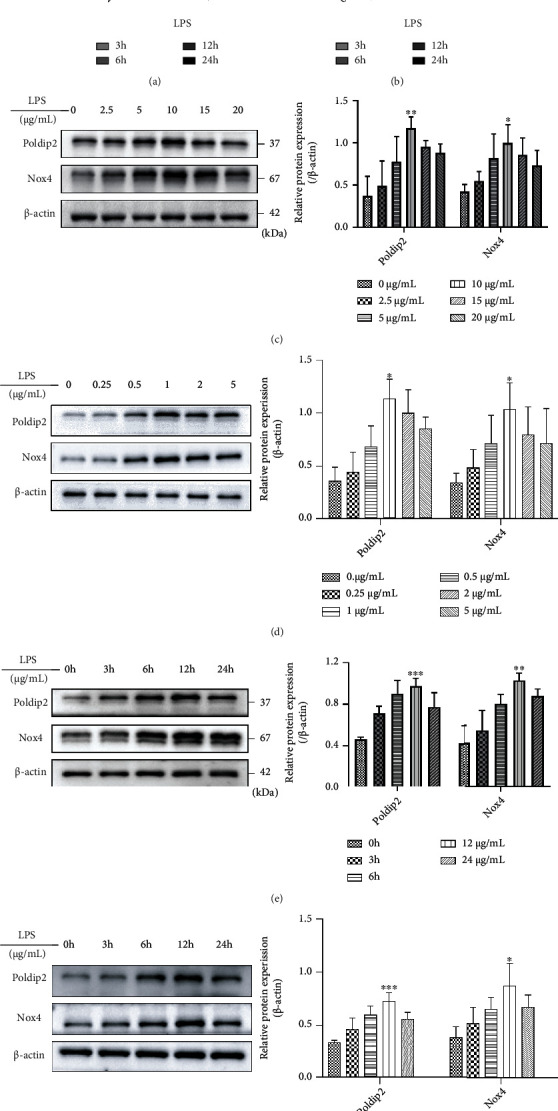
Poldip2 and Nox4 were upregulated in response to LPS in a dose/time-dependent manner in human lung epithelial cells. (a, b) A549 and Beas-2B cells were treated with the different doses of LPS (0–40 *μ*g/mL for A549 cells, 0–10 *μ*g/mL for Beas-2B cells) for 24 h, and the cell viability was analyzed by MTT assay. (c, d) Confluent cells were treated with different doses of LPS (0–20 *μ*g/mL for A549 cells and 0–5 *μ*g/mL for Beas-2B cells) for 12 h, and the protein levels of Poldip2 and Nox4 were detected by Western blotting and quantitatively analyzed. (e, f) Confluent cells were treated with LPS (10 *μ*g/mL for A549 cells and 1 *μ*g/mL for Beas-2B cells) for the different times (0–24 h), and the protein levels of Poldip2 and Nox4 were detected by Western blotting and quantitatively analyzed. Bars are mean ± standard deviation(SD) of 3 independent experiments. ^∗∗∗^*P* < 0.001 vs. 0 *μ*g/mL or 0 h, ^∗∗^*P* < 0.01 vs. 0 *μ*g/mL or 0 h, and ^∗^*P* < 0.05 vs. 0 *μ*g/mL or 0 h.

We first checked the effect of LPS stimulation on the viability of A549 and Beas-2B cells. Cells were exposed to the different doses of LPS (0–40 *μ*g/mL for A549 cells and 0–10 *μ*g/mL for Beas-2B cells) for 24 h in a serum-free medium, and cell viability was assessed by the MTT reduction assay. As LPS doses are rising, the cell viability had decreased significantly. As shown in Figures 1(a) and 1(b), for A549 cells, LPS at 40 *μ*g/mL significantly decreased cellular MTT reduction, but at 20 *μ*g/mL or less, it showed no toxicity. For Beas-2B cells, LPS at 10 *μ*g/mL notably decreased cellular MTT reduction, but at 5 *μ*g/mL or less, it showed no toxicity. Therefore, we chose to treat the cells with different doses of LPS (0–20 *μ*g/mL for A549 cells and 0–5 *μ*g/mL for Beas-2B cells) for 12 h in the following experiments.

Next, our study determined whether LPS affects the expression of Poldip2 and Nox4 and cells were exposed to different doses of LPS (0–20 *μ*g/mL for A549 cells and 0–5 *μ*g/mL for Beas-2B cells) for 12 h and protein expression was detected by Western blotting. In the two epithelial cells, the baseline expression of Poldip2 and Nox4 was at 0 *μ*g/mL and peaked at 10 *μ*g/mL and 1 *μ*g/mL, respectively (Figures 1(c) and 1(d)). Then, the protein levels of Poldip2 and Nox4 were monitored at the different times (0–24 h) after LPS treatment with 10 *μ*g/mL and 1 *μ*g/mL, respectively. The result showed that LPS significantly increased the expression of Poldip2 and Nox4 in a dose-dependent manner and the maximum levels were at 10 *μ*g/mL and 1 *μ*g/mL, respectively (Figures 1(e) and 1(f)). Taken together, LPS induced Poldip2 and Nox4 expression in a dose-/time-dependent manner in A549 and Beas-2B cells.

## 4. Poldip2 Immunoprecipitated with Nox4 in Response to LPS

**Figure 2:**
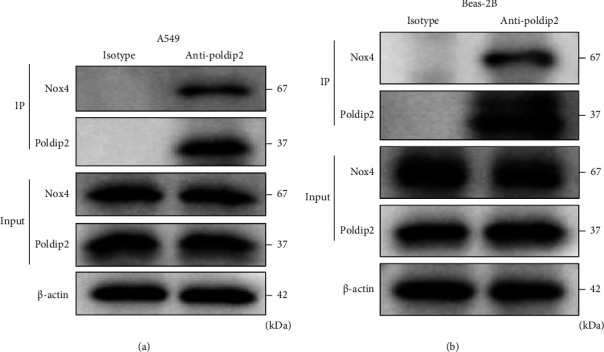
Poldip2 interacted with Nox4 induced by LPS with coimmunoprecipitation. (a, b) Confluent cells were treated with LPS (10 *μ*g/mL for A549 cells and 1 *μ*g/mL for Beas-2B cells) for 12 h. Cell lysates were immunoprecipitated with normal rabbit IgG (isotype controls) or primary rabbit anti-Poldip2 antibody and immunoblotted with a mouse anti-Nox4 antibody or rabbit anti-Poldip2 antibody. Lysis buffer (Input) is a positive control in coimmunoprecipitation.

Coimmunoprecipitation assay was used to confirm the interaction between Poldip2 and Nox4 during LPS stimulation. Using an antibody specific to Nox4, we observed that Poldip2 efficiently immunoprecipitated with Nox4 during LPS stimulation. The respective antibodies recognized two protein bands with expected sizes for Poldip2 (~37 kDa) and Nox4 (~67 kDa) in crude lysate-positive controls (input) (Figure 2).

### 4.1. Downregulation of Nox4 Suppressed LPS-Induced Oxidative Stress and Inflammation But Had No Effects on Poldip2 Expression

**Figure 3:**
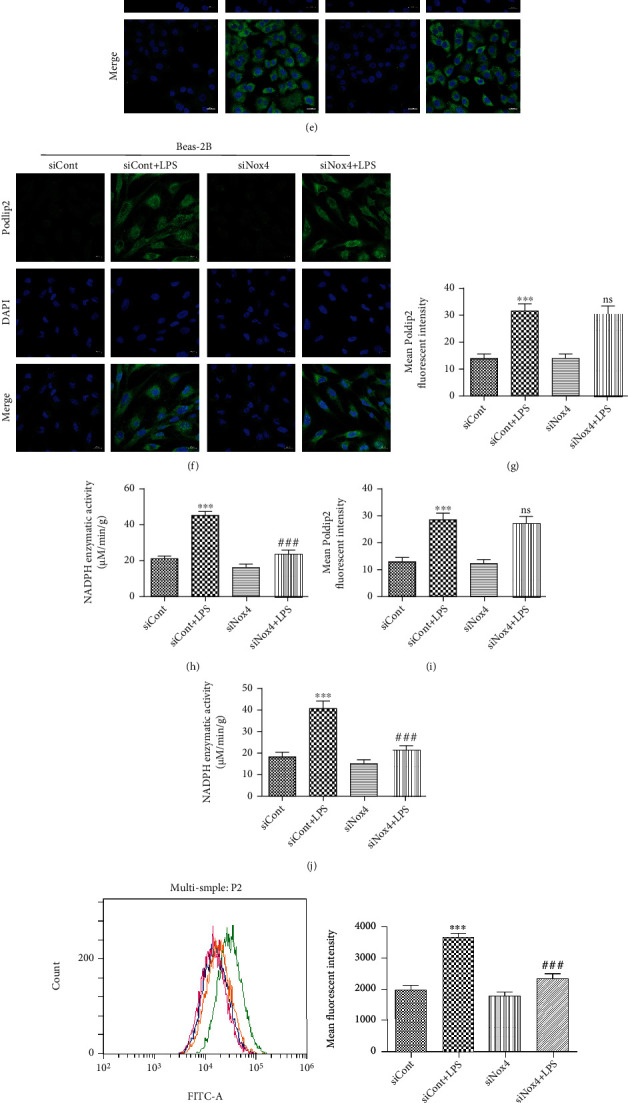
Nox4 is required for Poldip2 to exert action in response to LPS. (a, b) Confluent A549 and Beas-2B cells were transfected with siRNA for 72 h, and the efficiency of Nox4 downregulation was verified by Western blotting and quantitatively analyzed. Following transfection, they were treated with LPS (10 *μ*g/mL for A549 cells and 1 *μ*g/mL for Beas-2B cells) for 12 h. (c, d) The protein levels of Poldip2, HO-1, and COX-2 were detected by Western blotting and quantitatively analyzed. (e–g, i) The expression of Poldip2 was detected by immunofluorescence analysis and laser confocal microscope. The cells were stained with Poldip2 (green) and counter stained with DAPI (blue) (magnification of 400x). (h, j) The NADPH enzymatic activity in cells was detected by spectrophotometer. (k, l) The production of intracellular ROS was detected by flow cytometry. (m–r) The expression levels of PGE2, TNF-*α*, and IL-1*β* in the supernatant were detected by ELISA. Bars are mean ± SD of 3 independent experiments. ns: not significant, ^∗∗∗^*P* < 0.001 vs. siCont, ^∗∗^*P* < 0.01 vs. siCont, ^∗^*P* < 0.05 vs. siCont, ^###^*P* < 0.001 vs. siCont + LPS, ^##^*P* < 0.01 vs. siCont + LPS, and ^#^*P* < 0.05 vs. siCont + LPS.

To further investigate the effect of Nox4 downregulation on LPS-induced oxidative stress and inflammation, we performed downregulation of Nox4 in A549 and Beas-2B cells. Cells were transfected with siRNA against Nox4 (siNox4) to downregulate Nox4 expression and compared with the control siRNA vector without construct (siCont) (Figures 3(a) and 3(b)). Transfected cells (siCont and siNox4) were treated with PBS or LPS for 12 h. HO-1 and COX-2, acting as the downstream targets of Nox4, were detected simultaneously, the results showed that downregulation of Nox4 markedly enhanced HO-1 expression but inhibited COX-2 expression (Figures 3(c) and 3(d)). Moreover, downregulation of Nox4 significantly inhibited the enzymatic activity of NADPH oxidases (Figures 3(h) and 3(j)), as well as ROS generation (Figures 3(k) and 3(l)) under LPS stimulation.

In addition, PGE2, the downstream effector of COX-2, and inflammatory cytokines (TNF-*α* and IL-1*β*) also were analyzed after LPS stimulation. The results showed that downregulation of Nox4 markedly decreased LPS-induced PGE2 production (Figures 3(m) and 3(o)) and the release of TNF-*α* (Figures 3(n) and 3(p)) and IL-1*β* (Figures 3(q) and 3(r)). However, downregulation of Nox4 had no effects on Poldip2 expression (Figures 3(c) and 3(d)), suggesting that Nox4 acted as the downstream of Poldip2 and may mediate the effect of Poldip2. Immunofluorescence analyses (Figures 3(e)–3(g) and 3(i)) were consistent with the results of Western blotting in Figures 3(c) and 3(d).

### 4.2. Upregulation of Nox4 Increased Oxidative Stress and Inflammation But Had No Effects on Poldip2 Expression

**Figure 4:**
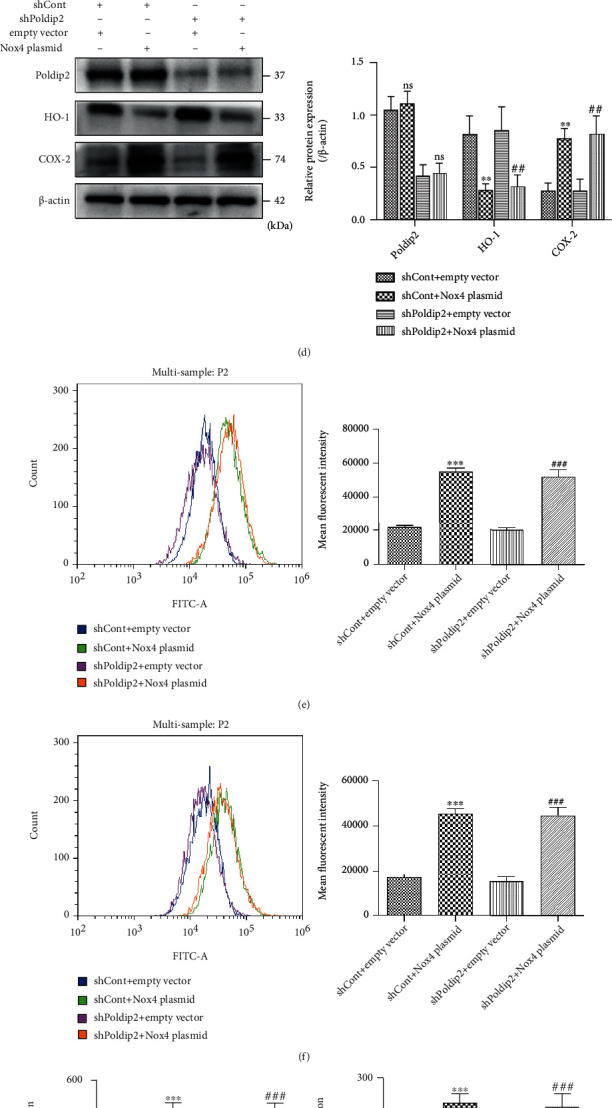
Upregulation of Nox4 increased oxidative stress and inflammation. (a, b) Confluent A549 and Beas-2B cells were transfected with Nox4 plasmid or the empty vector for 72 h, and the efficiency of Nox4 upregulation was verified by Western blotting and quantitatively analyzed. Confluent shCont and shPoldip2 cells were cotransfected with Nox4 plasmid or the empty vector for 72 h. (c, d) The protein levels of Poldip2, HO-1, and COX-2 were detected by Western blotting and quantitatively analyzed. (e, f) The production of intracellular ROS was detected by flow cytometry. (g–l) The expression levels of PGE2, TNF-*α*, and IL-1*β* in the supernatant were detected by ELISA. Bars are mean ± SD of 3 independent experiments. ^∗∗∗^*P* < 0.001 vs. shCont + empty vector, ^∗∗^*P* < 0.01 vs. shCont + empty vector, ^∗^*P* < 0.05 vs. empty vector, ^###^*P* < 0.001 vs. shPoldip2 + empty vector, and ^##^*P* < 0.01 vs. shPoldip2 + empty vector.

Furthermore, to explore the interdependence and causality of Poldip2 and Nox4, we performed the upregulation of Nox4 via plasmid transfection in the presence or absence of shPoldip2 in A549 and Beas-2B cells. A549 and Beas-2B cells were transfected with Nox4 plasmid or an empty vector for 72 h and the results showed that transfection of Nox4 plasmid significantly upregulated the Nox4 protein level comparing with the empty vector (Figures 4(a) and 4(b)). Then, transfected cells (shCont and shPoldip2) were cotransfected with Nox4 plasmid or the empty vector for 72 h. Our data suggested that, compared with cells transfected with the empty vector, Nox4 upregulation markedly attenuated HO-1 expression but enhanced COX-2 expression (Figures 4(c) and 4 (d)), whereas it had no obvious effect on Poldip2. Accordingly, the production of ROS, PGE2, TNF-*α*, and IL-1*β* was also increased by Nox4 upregulation (Figures 4(e)–4(l)). Collectively, our study confirmed that Poldip2 acts upstream of Nox4 in lung epithelial cells, but whether Poldip2 functionally regulates NADPH enzymatic activity needs further research.

### 4.3. Effect of Poldip2 on LPS-Induced Oxidative Stress and Inflammation

**Figure 5:**
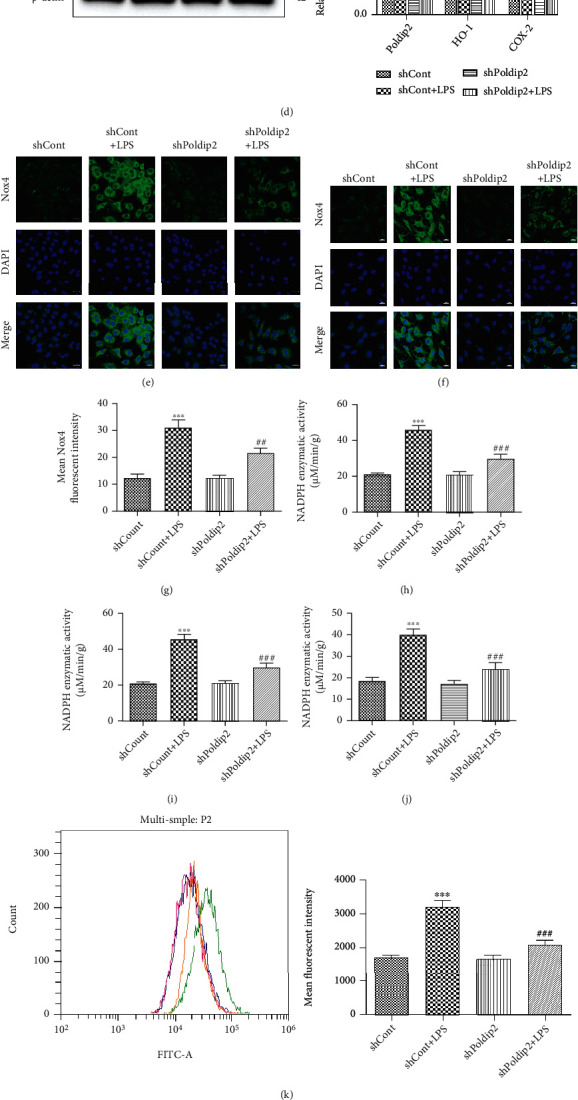
Downregulation of Poldip2 decreased LPS-induced oxidative stress and inflammation. (a, b) Confluent A549 and Beas-2B cells were transfected with the lentivirus downregulation vector for 72 h, and the efficiency of Poldip2 downregulation was verified by Western blotting and quantitatively analyzed. Following transfection, they were treated with LPS (10 *μ*g/mL for A549 cells and 1 *μ*g/mL for Beas-2B cells) for 12 h. (c, d) The protein levels of Nox4, HO-1, and COX-2 were detected by Western blotting and quantitatively analyzed. (e–g, i) The expression of Nox4 in the transfected cells was detected by immunofluorescence analysis and laser confocal microscope. The cells were stained with Nox4 (green) and counter stained with DAPI (blue) (magnification of 400x). (h, j) The NADPH enzymatic activity in cells was detected by a spectrophotometer. (k, l) The production of intracellular ROS was detected by flow cytometry. (m–r) The expression levels of PGE2, TNF-*α*, and IL-1*β* in the supernatant were detected by ELISA. Bars are mean ± SD of 3 independent experiments. ^∗∗∗^*P* < 0.001 vs. shCont, ^∗∗^*P* < 0.01 vs. shCont, ^∗^*P* < 0.05 vs. shCont, ^###^*P* < 0.001 vs. shCont + LPS, ^##^*P* < 0.01 vs. shCont + LPS, and ^#^*P* < 0.05 vs. shCont + LPS.

To further investigate the role of Poldip2 in LPS-induced oxidative stress and inflammation, we transfected A549 and Beas-2B cells with lentiviruses harboring short hairpin RNA (shRNA) against Poldip2 (shPoldip2) to downregulate Poldip2 expression and compared with the control lentivirus vector without construct (shCont) (Figures 5(a) and 5(b)). Transfected cells (shCont and shPoldip2) were treated with PBS or LPS for 12 h. We found that downregulation of Poldip2 via shPoldip2 markedly inhibited LPS-induced increases in the expression of Nox4 (Figures 5(c) and 5(d)), enzymatic activity of NADPH oxidases (Figures 5(h) and 5(j)), and ROS generation (Figures 5(k) and 5(l)). Immunofluorescence analyses (Figures 5(e)–5(g) and 5(i)) were consistent with the results of Western blotting in Figures 5(c) and 5(d). Meanwhile, downregulation of Poldip2 resulted in markedly enhanced HO-1 expression but attenuated COX-2 expression (Figures 5(c) and 5(d)) under LPS stimulation. Accordingly, the production of PGE2, TNF-*α*, and IL-1*β* was also decreased by Poldip2 downregulation (Figures 5(m)–5(r)).

**Figure 6:**
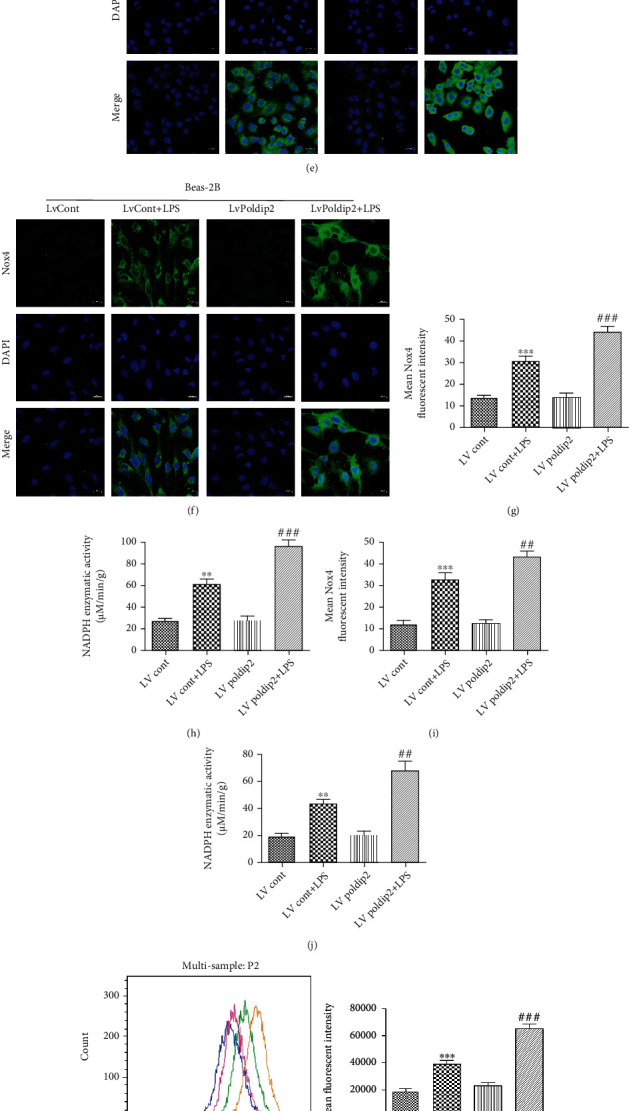
Upregulation of Poldip2 increased LPS-induced oxidative stress and inflammation. (a, b) Confluent A549 and Beas-2B cells were transfected with the lentivirus upregulation vector for 72 h, and the efficiency of Poldip2 upregulation was verified by Western blotting and quantitatively analyzed. Following transfection, they were treated with LPS (10 *μ*g/mL for A549 cells and 1 *μ*g/mL for Beas-2B cells) for 12 h. (c, d) The protein levels of Nox4, HO-1, and COX-2 were detected by Western blotting and quantitatively analyzed. (e–g, i) The expression of Nox4 in the transfected cells was detected by immunofluorescence analysis and a laser confocal microscope. The cells were stained with Nox4 (green) and counter stained with DAPI (blue) (magnification of 400x). (h, j) The NADPH enzymatic activity in cells was detected by a spectrophotometer. (k, l) The production of intracellular ROS was detected by flow cytometry. (m–r) The expression levels of PGE2, TNF-*α*, and IL-1*β* in the supernatant were detected by ELISA. Bars are mean ± SD of 3 independent experiments. ^∗∗∗^*P* < 0.001 vs. LvCont, ^∗∗^*P* < 0.01 vs. LvCont, ^∗^*P* < 0.05 vs. LvCont, ^###^*P* < 0.001 vs. LvCont + LPS, ^##^*P* < 0.01 vs. LvCont + LPS, and ^#^*P* < 0.05 vs. LvCont + LPS.

Conversely, we transfected lentivirus-upregulating Poldip2 particles to upregulate the Poldip2 expression. The results showed that Poldip2 upregulation (LvPoldip2) caused a significant increase of the Poldip2 protein level comparing with the control (LvCont) (Figures 6(a) and 6(b)). Then, transfected cells (LvCont and LvPoldip2) were treated as mentioned above. We also found that upregulation of Poldip2 exaggerated LPS-induced increases in the expression of Nox4 (Figures 6(c) and 6(d)), enzymatic activity of NADPH oxidases (Figures 6(h) and 6(j)), and ROS generation (Figures 6(k) and 6(l)). Immunofluorescence analyses (Figures 6(e)–6(g) and 6(i)) were consistent with the results of Western blotting in Figures 6(c) and 6(d). Meanwhile, upregulation of Poldip2 resulted in markedly attenuated HO-1 expression but enhanced COX-2 expression (Figures 6(c) and 6(d)) under LPS stimulation. Similarly, Poldip2 upregulation significantly exaggerated the LPS-induced increase in PGE2, TNF-*α*, and IL-1*β* production (Figures 6(m)–6(r)). Collectively, these data indicated that Poldip2 might be responsible for LPS-induced oxidative stress and inflammation via mediated Nox4 activation.

### 4.4. Role of Poldip2/Nox4-Mediating Oxidative Stress and Inflammation Was Dependent on PI3K-AKT Signaling

**Figure 7:**
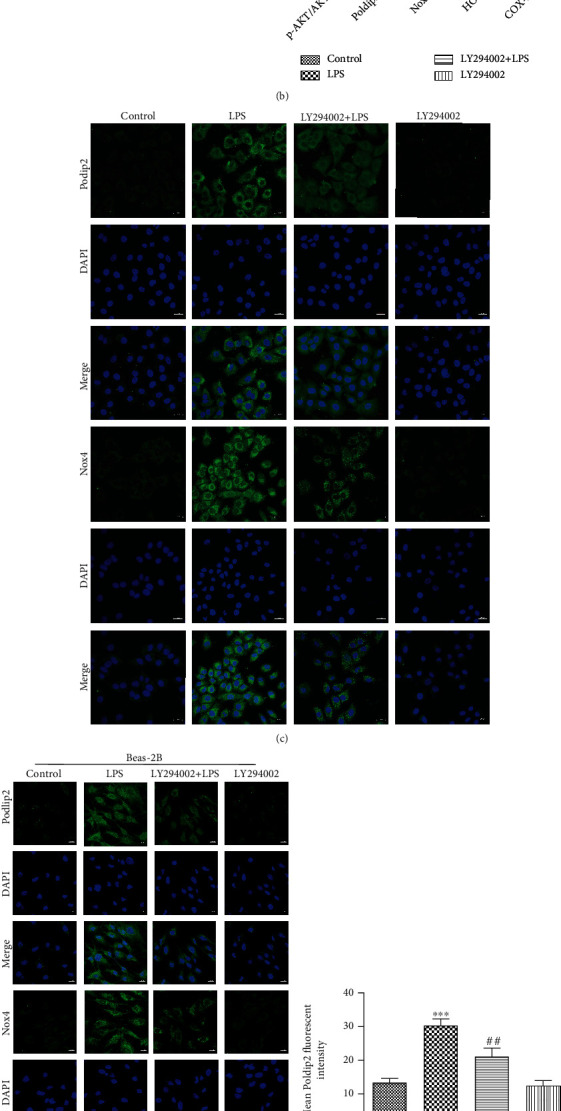
Effects of PI3K-AKT signaling on the activation of the Poldip2/Nox4-mediated pathway. Confluent A549 and Beas-2B cells were pretreated with 10 *μ*mol/L LY294002 for 1 h followed by postincubation with LPS (10 *μ*g/mL for A549 cells and 1 *μ*g/mL for Beas-2B cells) for 12 h. (a, b) The protein levels of p-AKT, AKT, Poldip2, Nox4, HO-1, and COX-2 were detected by Western blotting and quantitatively analyzed. (c–h) The expression of Poldip2 and Nox4 in cells was detected by immunofluorescence analysis and a laser confocal microscope. The cells were stained with Poldip2 (green), Nox4 (green), and counter-stained with DAPI (blue) (magnification of 400x). Bars are mean ± SD of 3 independent experiments. ^∗∗∗^*P* < 0.001 vs. control, ^∗∗^*P* < 0.01 vs. control, ^∗^*P* < 0.05 vs. control, ^###^*P* < 0.001 vs. LPS, ^##^*P* < 0.01 vs. LPS, and ^#^*P* < 0.05 vs. LPS.

As previously mentioned, Poldip2 interacted with Nox4 in response to LPS stimulation and acted as a vital regulator of Nox4. PI3K-AKT signaling has been proven to increase the expression of NADPH oxidases via upregulating its regulatory subunit in mammalian cells [21]. Here, we further explored a role for the PI3K-AKT signaling in the activation of Poldip2/Nox4 in response to LPS and cells were pretreated with the PI3K inhibitor LY294002 (10 *μ*mol/L, final concentration, dissolved in DMSO) for 1 h followed by postincubation with LPS for 12 h. As shown in Figures 7(a) and 7(b), we determined the activation of PI3K-AKT signaling by analyzing the phosphorylation of its key effector, p-AKT, via Western blotting. LPS stimulation significantly increased the p-AKT expression, which was partially suppressed by pretreatment with LY294002. Importantly, the results showed that inhibition of PI3K resulted in a reduction in LPS-induced increases in Poldip2, Nox4, and COX-2, as well as decreases in HO-1 expression. Immunofluorescence analyses (Figures 7(c)–7(h)) were consistent with the results of Western blotting in Figures 7(a) and 7(b). Collectively, these data above suggest that PI3K-AKT signaling is required for the LPS-mediated Poldip2/Nox4 activation.

## 5. Discussion

In this study, our data demonstrated that LPS induced oxidative stress and inflammation in lung epithelial cells by increasing the expression of Nox4 and COX-2 and NADPH enzymatic activity and substantially increasing the production of ROS, PGE2, TNF-*α*, and IL-1*β* and decreasing the HO-1 expression. Remarkably, Poldip2, a novel regulator of Nox4, interacted with Nox4 and acted as an upstream regulator of Nox4 in LPS-stimulated lung epithelial cells. By downregulation or upregulation of Poldip2, we confirmed that Nox4 requires Poldip2 for ROS generation and subsequent inflammation. Taken together, our findings reveal that Poldip2 mediates oxidative stress and inflammation via interaction with Nox4 and such effect of Poldip2/Nox4 is regulated by the PI3K-AKT signaling in LPS-stimulated lung epithelial cells. Our results suggest that Poldip2 may be a potential therapeutic candidate for the treatment of ALI.

It is well known that LPS stimulation can induce excessive ROS generation that exaggerated a range of respiratory inflammatory diseases, such as chronic obstructive pulmonary disease (COPD), asthma, and ALI [22]. NADPH oxidase was an important source for the generation of ROS [23]. Distinct from other members of the NADPH oxidase, Nox4 was constitutively active and played a detrimental role in the development of inflammatory diseases [24, 25]. Both A549 and Beas-2B cells are commonly used to investigate the cellular mechanism of lung inflammation under LPS stimulation, including oxidative stress and autophagy [26, 27]. Herein, our results showed clearly that LPS significantly increased NOX4 expression in lung epithelial cells in dose-/time-dependent manners, which were paralleled to the expression of Poldip2. Over the years, Poldip2 is emerging as an important mediator in inflammatory diseases, including sepsis-associated encephalopathy [28] and ARDS [13]. Previous findings have demonstrated that heterozygous deletion of Poldip2 or Nox4 knockdown markedly reduces superoxide production and improves survival in the ALI/ARDS mouse model [13, 23]. All these results suggested the possibility that coincident expression of Poldip2 and Nox4 was essential for the progression of ALI.

Previous data suggested that Poldip2 binds to Nox4 and showed a functional association in vascular smooth muscle [20]; in line with this, we found a correlation between Poldip2 and Nox4 in the regulation of LPS-induced oxidative stress and inflammation in lung epithelial cells. We further explored how Poldip2 and Nox4 interacted by using the siRNA or upregulation of plasmid targeting Nox4. Our data suggested that Nox4 acted as the downstream of Poldip2 and was required for the effect of Poldip2 in response to LPS. consistent with our finding, Datla and his colleagues showed that Poldip2 controls Vascular Smooth Muscle Cell (VSMC) migration through regulating focal adhesion turnover and traction force production via activation of Nox4/RhoA/FAK signaling [29]. However, as we know, the downstream targets of Nox4 implicated in the development of ALI have not been well understood. HO-1, an inducible antioxidant enzyme, is considered to be the main protein protecting against LPS-induced ALI [30]. Meanwhile, COX-2, a proinflammatory mediator, is highly expressed in alveolar epithelial cells and its expression contributes to the poor outcome of ALI [31]. It has been reported that LPS-induced HO-1 and COX-2 expression was significantly attenuated by NADPH oxidase inhibitors [32, 33]. Consistent with these studies, we found that downregulation of Nox4 using siRNA significantly suppressed COX-2 expression but enhanced HO-1 expression under LPS stimulation.

The NADPH oxidase enzyme is an enzyme complex system, which composes of transmembrane protein catalytic subunits and cytosolic regulatory subunits; it becomes active after regulatory subunits translocate to the plasma membrane and interact with catalytic subunits to form the functional enzyme [34]. However, the mechanism of Nox4 activation in response to LPS has not been fully understood. As described earlier, Poldip2 as acted an upstream regulator of Nox4 and interacted with Nox4 under LPS stimulation. Importantly, after lentivirus-mediated Poldip2 downregulation and being challenged by LPS in lung epithelial cells, we found that the expression of Nox4, enzymatic activity of NADPH oxidases, and production of ROS were significantly inhibited, which were accompanied by decreased COX-2 and increased HO-1 expression, indicating that Poldip2 may function similarly to other NADPH oxidase cytosolic regulatory subunits. Indeed, upregulation of Poldip2 significantly increased oxidative stress and inflammation in LPS-stimulated lung epithelial cells. Consistent with our findings, a previous study also supported the active effects of Poldip2 on Nox4 expression. For example, upregulation of Poldip2 using adenovirus caused a significant increase in Nox4 enzymatic activity and basal ROS production, whereas Poldip2 downregulation by siRNA significantly decreases ROS production in VSMCs [20]. It is therefore possible that inhibition of Poldip2 expression may represent a novel mechanism for limiting oxidative stress and inflammation in LPS-induced ALI.

In addition, PI3K-AKT signaling was considered as a critical signaling pathway in mammalian cells that regulates cell growth, proliferation, metabolism, and motility [35]. Increasing evidence has suggested that PI3K-AKT signaling is a major pathway involved in the development of ALI [36, 37]. In our study, we explored that pretreatment with LY294002 significantly suppressed LPS-induced increase in Poldip2, Nox4, and COX-2 and decrease in HO-1 in lung epithelial cells, indicating that PI3K-AKT signaling acts as an upstream regulator of Poldip2. We speculated that PI3K-AKT signaling may be involved in regulating Poldip2 expression and eventually affected the activity of Nox4. However, the precise mechanism involved in PI3K-AKT signaling that regulates Poldip2 expression is unclear and requires further investigation.

Our findings suggest that Poldip2 is involved in oxidative stress and inflammation through interacting with Nox4 and is regulated by the PI3K-AKT signaling, which may contribute to better understanding of the plausible mechanism of acute lung injury.

## Data Availability

The data used to support the findings of this study are available from the corresponding author upon request.
